# Citrullination as early-stage indicator of cell response to Single-Walled Carbon Nanotubes

**DOI:** 10.1038/srep01124

**Published:** 2013-01-24

**Authors:** Bashir Mustafa Mohamed, Dania Movia, Anton Knyazev, Dominique Langevin, Anthony Mitchell Davies, Adriele Prina-Mello, Yuri Volkov

**Affiliations:** 1 Department of Clinical Medicine, Trinity College Dublin, Ireland; 2 Centre for Research on Adaptive Nanostructures and Nanodevices, Trinity College Dublin, Ireland; 3 Universite Paris 11-CNRS, Laboratoire de Physique des Solides, France; 4 These authors contributed equally to this work.

## Abstract

Single-walled carbon nanotubes (SWCNTs) have been widely explored as potential technologies for information systems and medical applications. The impact of SWCNTs on human health is of prime concern, if SWCNTs have a future in the manufacturing industry. This study proposes a novel, inflammation-independent paradigm of toxicity for SWCNTs, identifying the protein citrullination process as early-stage indicator of inflammatory responses of macrophages (THP-1) and of subtle phenotypic damages of lung epithelial (A549) cells following exposure to chemically-treated SWCNTs. Our results showed that, while most of the cellular responses of A549 cells exposed to SWCNTs are different to those of similarly treated THP-1 cells, the protein citrullination process is triggered in a dose- and time-dependent manner in both cell lines, with thresholds comparable between inflammatory (THP-1) and non-inflammatory (A549) cell types. The cellular mechanism proposed herein could have a high impact in predicting the current risk associated with environmental exposure to SWCNTs.

Single-walled carbon nanotubes (SWCNTs) represent a relatively newly discovered allotrope of carbon^1^ having a wide range of potential applications in the information and communications technology (ICT)^2^,^3^ and medicine^4^,^5^,^6^,^7^ fields.

As for many other nanomaterials, several aspects of the effects of SWCNTs to human health are poorly defined^8^. From 2006 onwards, the number of reports investigating *in vitro*^9^,^10^ and *in vivo*^11^,^12^,^13^ the lung toxicity of SWCNTs has increased exponentially but the current knowledge of SWCNTs' adverse effects in the lungs is still fragmentary, and sometimes contradictory^8^,^14^,^15^. This fragmentation originates from three main aspects^16^. Firstly, the studies exploring SWCNTs' toxicity are not standardised for SWCNTs produced by different methods and do not employ equivalent testing models. Secondly, in many cases such experiments do not accurately reflect actual exposure conditions. This originates ultimately from the absence of environmental exposure data on SWCNTs and of specific information regarding the end-of-life/disposal of SWCNTs-based devices from ICT and medical applications. Thirdly, specific cellular mechanisms through which SWCNTs may exert their adverse toxic effects *in vivo* are still under debate.

By looking at the emerging picture, the most widely accepted paradigms^14^ for SWCNTs' toxicity are (a) the oxidative stress^13^,^17^,^18^ (due to redox features of this nanomaterial^19^ or of transition metals and other environmental contaminants present in SWCNTs^18^,^20^) and (b) inflammation (associated with inflammatory and fibrogenic responses in the lungs^21^ and ineffective recognition of SWCNTs by macrophages^22^). This study proposes a potential novel paradigm according to which SWCNTs triggered a toxic response by activating an inflammation-independent cellular mechanism. Citrullination (i.e., the enzymatic conversion of protein-contained arginine to citrulline carried out by peptidylarginine deiminase (PAD)) was identified as an early-stage indicator of such inflammation-independent mechanism. Macrophages (THP-1 cells) and lung epithelial (A549) cells were exposed to chemically-treated SWCNTs and the protein citrullination levels were quantified. Cellular protein modification levels were then correlated to cytotoxic cellular responses and to the secretion of pro-inflammatory cytokines.

Citrullination is known as an inflammation associated process^23^ characteristic of extra-articular manifestations of rheumatoid arthritis (RA)^24^,^25^,^26^, such as interstitial pneumonia and rheumatoid nodules. Our group has recently demonstrated that exposure to various nanomaterials does induce citrullination^27^, which might ultimately trigger autoimmune diseases. Protein citrullination, as well as Ca^2+^-mediated PAD activation, was in fact detected in cultured human cells (A549 and THP-1 cells) and in mouse lung tissue after exposure to nanosized amorphous silicon dioxide (SiO_2_), ultra-fine carbon black (ufCB) and SWCNTs^27^.

Here, we present for the first time evidence that SWCNTs are able to induce protein citrullination in a lung epithelial cell line prior to any detectable onset of inflammatory responses. SWCNTs were tested following chemical treatment, since chemical modifications are known to reduce the amount of toxic metal impurities (used in the production of the carbon nanomaterials and present in the as-produced, pristine SWCNTs) that can ultimately induce oxidative stress and cytokine secretion in cells exposed to SWCNTs. Furthermore, chemical modifications are fundamental to increasing the manufacturability and biocompatibility of SWCNTs for ICT and medical applications, thus making our testing model relevant when compared to realistic exposure scenarios of the manufacturing industry. Our results clearly indicated that exposure to SWCNTs induced citrullination in THP-1 and A549 cells after 6 h. In particular, high citrullination levels were detected in lung epithelial cells, which are not responsible for inflammation responses, thus ultimately advocating citrullination as an inflammation-independent process and highlighting the potential use of citrullination as early-stage indicator of an emerging inflammation-independent paradigm of SWCNTs' toxicity.

## Results

### High content screening and analysis (HCSA)

#### Protein citrullination

**Figure 1:**
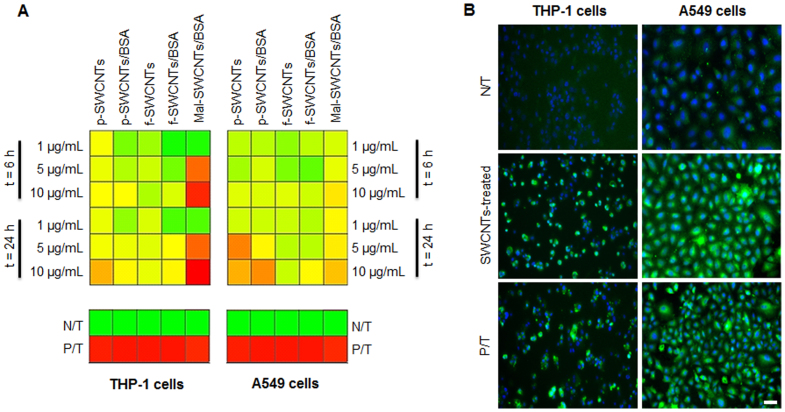
Citrullination levels in THP-1 and A549 cells exposed to chemically-treated SWCNTs (p-SWCNTs, p-SWCNTs/BSA, f-SWCNTs, f-SWCNTs/BSA and Mal-SWCNTs/BSA) for 6 and 24 h. (A) Graphical tables (heatmaps) reflect the citrullination levels ranging from dark green (lower than 15% change from the maximum value measured) to bright green (30%), yellow (50%), bright orange (60%), dark orange (75%) and finally red (higher than 75% change from the maximum value). (B) Representative fluorescent images of untreated (N/T), SWCNTs-treated and PAD-treated (P/T) THP-1 and A549 cells after 24 h exposure. Cells were immunostained for citrulline expression (in green) and nuclei (in blue). SWCNTs sample: p-SWCNTs; scale bar: 40 μm (10× magnification).

Human lung epithelial (A549) cells and phagocytic (THP-1) cells were exposed to various SWCNTs concentrations (1, 5, 10 μg/ml) for 6 h and 24 h, and HCSA was carried out to quantify the induction of protein citrullination. The data (Figure 1A) indicated a significant increase in protein citrullination in both cell lines in a time- and dose-dependent manner, with maximum effects seen at 24 h and at the highest dose (10 μg/ml) tested. Immunofluorescent images of cells exposed to chemically-treated SWCNTs (Figure 1B) showed citrullinated proteins in the cytoplasm and around the nucleus after 24 exposure, thus validating that citrullinated proteins were expressed.

By increasing the complexity of the SWCNT's functionalization (p-SWCNTs < p SWCNTs/BSA < f-SWCNTs < f-SWCNTs/BSA), the induction of citrullinated proteins decreased. High citrullination levels were found in fact when cells were treated with p-SWCNTs, while cells exposed to f-SWCNTs/BSA showed low citrullination levels. However, high levels of citrullination were detected after exposure to Mal-SWCNTs/BSA. This could be associated with the presence of cytotoxic maleic moieties on the nanotubes surface.

Interestingly, significantly high citrullination was observed in A549 cells (which are cells not directly involved in inflammation responses) treated with SWCNTs. In detail, citrullination was detectable in SWCNTs-treated A549 cells even after 6 h exposure (Figure 1A). This suggested that the induction of protein citrullination could be considered as a sign of early cellular damage in an *in vitro,* non-inflammatory model such as A549 cells.

#### Cytotoxicity responses

**Figure 2:**
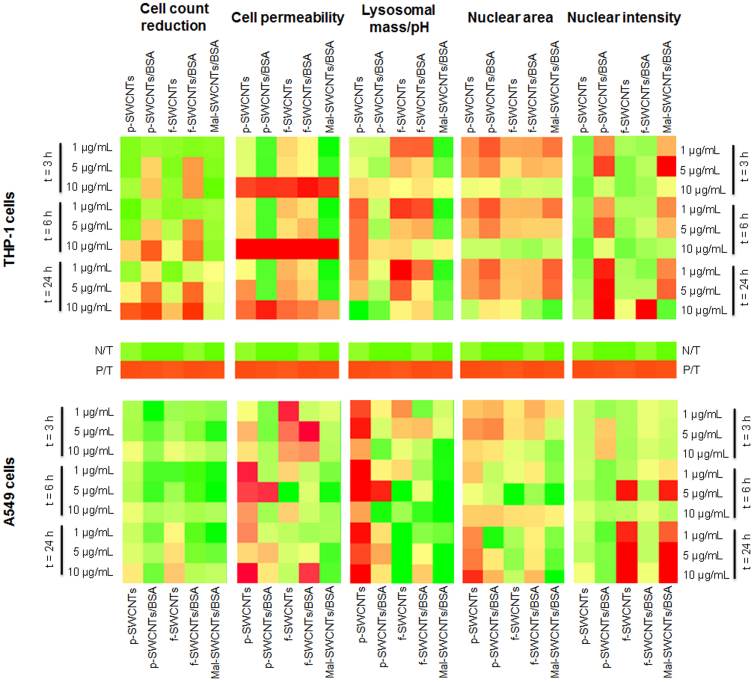
Graphical tables (heatmaps) of the cytotoxicity data of THP-1 and A549 cells exposed to p-SWCNTs, p-SWCNTs/BSA, f-SWCNTs, f-SWCNTs/BSA and Mal-SWCNTs/BSA for 3, 6 and 24 h. Colorimetric gradient tables reflect the changes in cell count reduction, cell membrane permeability, lysosomal mass/pH, nuclear area and nuclear intensity. Colours range from dark green (values lower than 15% change from the maximum value measured) to bright green (30%), yellow (50%), bright orange (60%), dark orange (75%) and finally to red (values higher than 75% change from maximum value). Heatmap values are normalised to the percentages of the positive control (P/C) and Z-score is calculated as described in the statistical analysis section. Data represent two independent experiments performed in triplicate samples.

Three different concentrations (1, 5, 10 μg/ml) of SWCNTs were tested on THP-1 and A549 cell populations at three time points (3, 6, 24 h). Cell count reduction, cell membrane permeability, lysosomal mass/pH and nuclear morphology changes were the cell parameters monitored by HCSA (Figure 2).

#### Cell count reduction

A dose- and time- dependent reduction of cell viability was detected when THP-1 cells were exposed to SWCNTs (Figure 2). In detail, significant reduction in cell count was seen after 3 h exposure to p-SWCNTS/BSA and f-SWCNTS/BSA, and after 6 h exposure to p-SWCNTs, f-SWCNTs and Mal-SWCNTs/BSA. In contrast, A549 cells showed a decreased cell count only when exposed to the highest concentration (10 μg/ml) of p-SWCNTs and f-SWCNTs for 24 h.

#### Cell membrane permeability

It has been shown that alterations of the cellular membrane permeability are often associated with an on-going toxic or apoptotic cell responses (21). A clear dose-dependent increase in cell membrane permeability was observed in both THP-1 and A549 cells when exposed to p-SWCNTs, f-SWCNTs and f-SWCNTs/BSA (Figure 2), whereas p-SWCNTs/BSA and Mal-SWCNTs/BSA caused significant cell membrane permeability changes only at the highest concentration (10 μg/ml) in both cell lines.

#### Lysosomal mass/pH

Lysosomal mass/pH changes were significant when THP-1 cells were exposed to p-SWCNTs, f-SWCNTs and f-SWCNTs/BSA, while subtle lysosomal mass/pH changes were detected in THP-1 cells exposed to p-SWCNTs/BSA and Mal-SWCNTs/BSA (Figure 2). Lysosomal mass/pH was also markedly altered in A549 cells, with the lowest response associated with the exposure to Mal-SWCNTs/BSA.

#### Nuclear area and nuclear intensity

Since changes in the nuclear morphology can be an additional sign of cell stress and apoptotic/toxic stimuli, the nuclear size and intensity of THP-1 and A549 cells were monitored after exposure to SWCNTs for extended exposure intervals (24 h). We observed significant nuclear morphological changes in SWCNTs-treated THP-1 cells, while the nuclear morphology was only marginally altered in A549 cells exposed to SWCNTs (Figure 2).

### Cytokines secretion

To further explore the possible interdependence between the citrullination process and the inflammatory changes in cells of different origin as triggered by exposure to chemically-treated SWCNTs, the secretion of the pro-inflammatory cytokines Tumour Necrosis Factor-alpha (TNF-α) and Interleukin-6 (IL-6) was quantified after treatment of inflammatory (THP-1 cells) and non-inflammatory (A549) cells with such nanomaterials.

**Figure 3:**
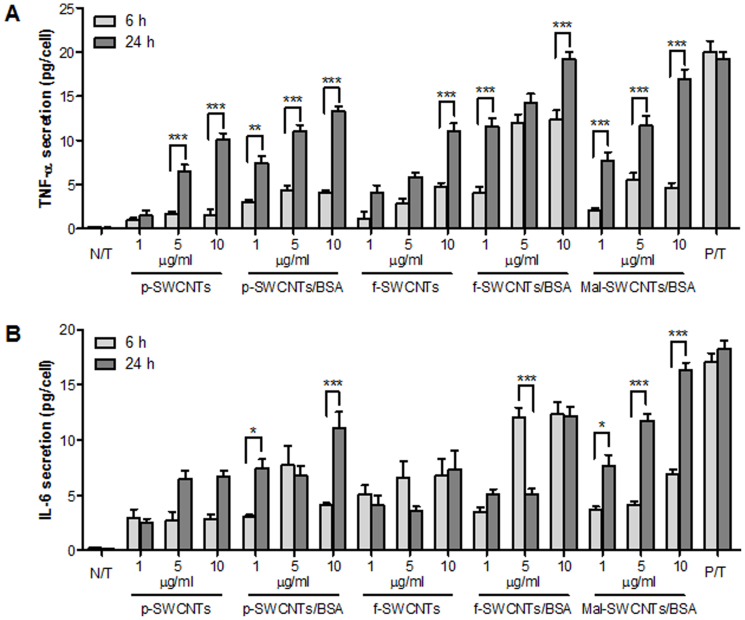
Release of (A) TNF-α and (B) IL-6 from THP-1 cells after 6 h (light grey) and 24 h (dark grey) exposure to p-SWCNTs, p-SWCNTs/BSA, f-SWCNTs, f-SWCNTs/BSA and Mal-SWCNTs/BSA at various concentrations (1, 5 and 10 μg/mL). The symbols (*), (**) and (***) indicate significant time-dependent changes (*p* < 0.05, *p* < 0.01 and *p* < 0.001, respectively).

**Figure 4:**
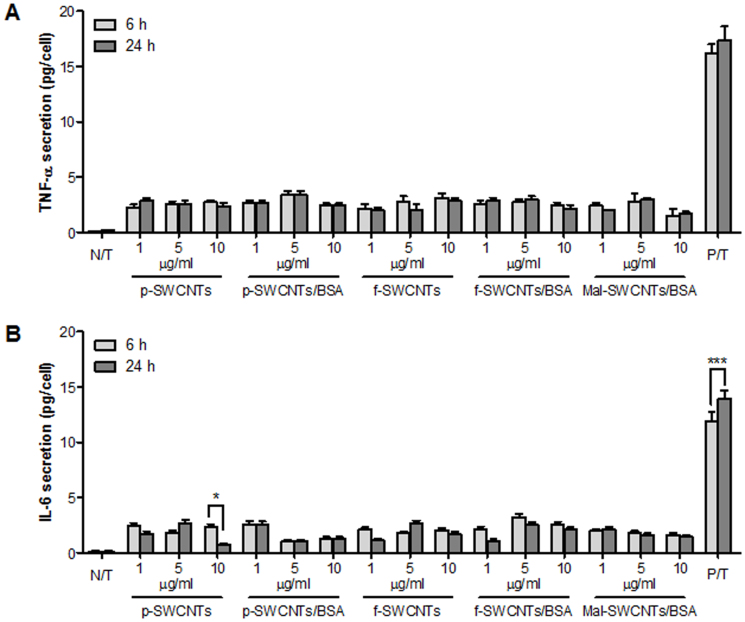
Release of (A) TNF-α and (B) IL-6 from A549 cells after 6 h (light grey) and 24 h (dark grey) exposure to p-SWCNTs, p-SWCNTs/BSA, f-SWCNTs, f-SWCNTs/BSA and Mal-SWCNTs/BSA at various concentrations (1, 5 and 10 μg/mL). The symbols (*) and (***) indicate significant time-dependent changes (*p* < 0.05 and *p* < 0.001, respectively).

TNF-α and IL-6 are known to be among the most important cell-signalling protein molecules secreted by activated macrophages (such as PMA-activated THP-1 cells) during inflammation^28^. Figure 3 and Figure 4 display the amount of TNF-α and IL-6 secreted by THP-1 and A549 cells exposed for 6 and 24 h to increasing concentrations (1, 5 and 10 μg/ml) of p-SWCNTs, p-SWCNTs/BSA, f-SWCNTs, f-SWCNTs/BSA, Mal-SWCNTs/BSA. For THP-1 cells, we observed that the secretion resulted to be time- and dose-dependent. Interestingly, a higher secretion of TNF-α and IL-6 was detected when SWCNTs were conjugated with BSA (p-SWCNTs/BSA, f-SWCNTs/BSA and Mal-SWCNTs/BSA), while lower levels of TNF-α and IL-6 were detected after exposure to unconjugated SWCNTs (p-SWCNTs and f-SWCNTs). This phenomenon could be associated with the higher de-bundling of BSA-conjugated SWCNTs, which might have triggered the onset of acute phase reaction.

In contrast, exposure of A549 cells to SWCNTs did not significantly trigger the secretion of TNF-α (Figure 4A) or IL-6 (Figure 4B) over 6 and 24 h exposure, leaving the levels of the secreted proteins comparable to those of the untreated cell culture (N/T).

## Discussion

Citrullination has been reported to be a process present in a wide range of inflammatory tissues, suggesting that this is an inflammation-dependent rather than disease-dependent process^23^. Our group has previously demonstrated that nanomaterials of different origin are capable of promoting specific transformation of the amino acid arginine into the molecule called citrulline^27^, which can lead ultimately to the development of autoimmune conditions such as rheumatoid arthritis (RA)^14^. Here, we showed for the first time that exposure to differently processed SWCNTs (in terms of the chemical treatment applied and functionalities/chemical compounds decorating their surface) significantly influenced the protein citrullination levels in cultured THP-1 cells as well as in non-inflammatory lung epithelial cells (Figure 1), thus suggesting citrullination as an early-stage marker of a novel inflammation-independent paradigm for SWCNTs toxicity.

THP-1 and A549 cells were selected as the most relevant *in vitro* models for our study. THP-1 cells were employed to represent the resident phagocytic cells that have the main function of removing pathogens, senescent cells and external particles from the lungs^29^,^30^, while A549 cells represented an *in vitro* model for lung alveoli cells, which are not associated with inflammatory responses. A549 cells have been extensively used for assessing pulmonary cytotoxicity, including nanomaterials-induced cytotoxicity^10^,^31^,^32^.

Our results showed that, following exposure to SWCNTs, high levels of citrullination were found in cultured A549 cells, as well as in THP-1 cells, independently of the chemical treatment applied to SWCNTs (Figure 1). Furthermore, we found that, even if protein citrullination occurred, the cytotoxic (Figure 2) and inflammatory (Figure 4) cellular responses were significantly lower in SWCNTs-treated A549 cells than in similarly treated THP-1 cells (Figure 2 and Figure 3, respectively). Similarly to a previous study published by Hu *et al.*^33^, showing that lymphocytes were more sensitive to SWCNTs as compared to human lung cells, our data proved that phagocytic THP-1 cells were highly sensitive to all SWCNTs samples (Figure 2), responding to the SWCNTs exposure with high levels of secreted pro-inflammatory cytokines (TNF-α and IL-6) (Figure 3A and Figure 3B, respectively). In contrast, A549 cells were only marginally affected by the exposure to the chemically-treated SWCNTs (Figure 2). Additional analysis of the inflammatory mediators did not show in fact any significance secretion of TNF-α and IL-6 from A549 cells exposed to SWCNTs (Figure 4). These results, together with literature data showing that SWCNTs can lead to the suppression of a variety of inflammatory mediators (including IL-6) in *in vitro* lung epithelial cell models^34^, provide evidence that SWCNTs could be possibly cytotoxic to lung epithelial cells by activating an inflammation-independent cellular mechanism. Keeping in mind that citrullination of proteins is a process distinct from the formation of the free amino acid citrulline as by-product of oxidative stress enzymes, our results suggest citrullination as a potential early-stage marker for a novel paradigm of SWCNTs' toxicity.

In conclusion, our study showed for the first time that increased increased protein citrullination can be a potential robust indicator for a novel inflammation-independent paradigm defining the SWCNTs lung toxicity. Given that exposure to SWCNTs triggered an inflammatory response of THP-1 cells, including markedly increased levels of secreted pro-inflammatory cytokines, one could assume that citrullination occurred as a down-stream process of the inflammatory response to SWCNTs. However, our results in a non-inflammatory, *in vitro* cell model (such as A549 cells) showed that protein citrullination was not necessarily an inflammation-dependent process. In detail, small changes in cytotoxic cellular responses and low levels of cytokines secretion were associated with relatively high levels of protein citrullination in A549 cells exposed to five different types of SWCNTs.

Our results strongly advocate for a novel inflammation-independent paradigm of SWCNTs toxicity and the use of citrullination levels as an early-stage indicator for the hazard ranking of SWCNTs and potentially of nanomaterials, more in general. Further studies are required to fully explore the mechanism(s) involved in the citrullination induced by exposure to SWCNTs *in vitro* and in *in vivo* correlation studies.

## Methods

### Materials

**Figure 5:**
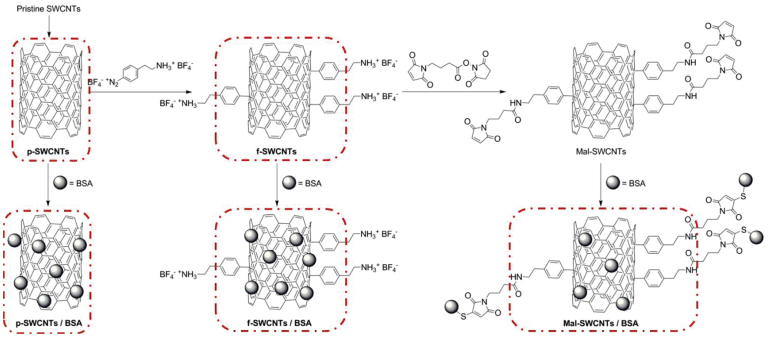
Schematic of the purification and covalent functionalization of pristine SWCNTs. Boxes highlight the SWCNTs samples tested in this study. Figure adapted from Knyazev *et al.*^35^.

Chemicals and solvents were purchased from commercial sources (Sigma-Aldrich, Fisher Scientific, Invitrogen and Calbiochem). Pristine SWCNTs prepared by laser ablation were purified and functionalized following the procedure reported in a previous study^35^ (Figure 5). Briefly, pristine SWCNTs were first purified (p-SWCNTs) and then functionalized by adding 4-(2-aminoethyl)benzenediazonium tetrafluoroborate, thus obtaining covalently functionalized single-walled carbon nanotubes (f-SWCNTs). The f-SWCNTs nanotubes were then treated with 4-maleimidobutyric acid N-hydroxysuccinimide ester, obtaining Mal-SWCNTs. The resulting p-SWCNTs, f-SWCNTs and Mal-SWCNTs derivatives were exposed to a solution of bovine serum albumin (BSA) in 4-(2-hydroxyethyl)-1-piperazineethanesulfonic acid (HEPES), thus yielding p-SWCNTs/BSA, f-SWCNTs/BSA and Mal-SWCNTs/BSA, respectively. Table S23 in the Supporting Information reports a summary of the characterization of the SWCNTs samples (average atomic force microscopy height measurements, O_2_ atomic percentage and zeta potential of SWCNT dispersions in water at pH 7).

### Cell culture

Human lung epithelial (A549) and phagocytic (THP-1) cell lines (ATCC, Manassas, VA, USA) were cultured in DMEM and RPMI 1640 medium, respectively. Both the media were supplemented with 10% (v/v) foetal bovine serum (FBS) and 1% (v/v) L-glutamine/penicillin/streptomycin. Cells were grown in a humidified incubator at 37°C in 5% CO_2_.

### Cell plating and exposure

For experimentation, A549 and THP-1 cells were seeded in 96-well plates at a concentration of 5,000 and 15,000 cells/well, respectively (Nunc Inc., USA). THP-1 cells were stimulated with 25 ng/ml of phorbol 12-myristate 13-acetate (PMA) for 72 h before exposure to SWCNTs. THP-1 and A549 cells were exposed to five different samples of chemically-treated SWCNTs, namely p-SWCNTs, p-SWCNTs/BSA, f-SWCNTs, f-SWCNTs/BSA and Mal-SWCNTs/BSA. Exposure concentrations ranged from 1 μg/ml to 10 μg/ml (1, 5 and 10 μg/ml).

### High content screening and analysis (HCSA)

HCSA combines high-resolution digital imaging with powerful software algorithms to enhance the quantitative data processing. HCSA is currently recognised as the industry standard for drug screening. Recently, HCSA has also been implemented for the analysis of cell changes induced by nanomaterials^27^,^36^,^37^,^38^,^39^ since this is the only contemporary technique which enables to handle large experimental data sets correlating cell responses to multiple nanomaterials, reagents, concentrations and data points. HCSA was used to quantify citrullination process and cytotoxic responses following exposure to SWCNTs.

- Protein citrullination: Cells were exposed to SWCNTs for 6 h and 24 h and then fixed using 3% paraformaldehyde (PFA), as previously described^27^. After gentle washing with phosphate buffer solution (PBS), cells were incubated with anti-citrulline antibody (Cat. No: ab6464, 1:200 dilution) for 1 h at room temperature. Cells were washed three times with PBS and then incubated with FITC-linked goat anti-rabbit antibody for 1 h and stained for nuclei with Hoechst 33342. Plates were scanned using IN Cell Analyzer 1000 automated microscope (GE Healthcare, Buckinghamshire, UK). Images were acquired in a stereology configuration of five randomly selected fields per well at 10× magnification using two detection channels. Protein citrullination was quantified using the dual area object analysis module of the IN Cell Investigator software (GE Healthcare, Buckinghamshire, UK). The module allows for simultaneous quantification of subcellular inclusions that are marked by different fluorescent labels and measures fluorescence intensity associated with predefined nuclear and cytoplasmic compartments. Cells exposed to peptidylarginine diminase (PAD) were used as positive control (P/T).
- Cytotoxicity assay: Following exposure to SWCNTs for 3, 6 and 24 h, a multiparametric cytotoxicity assay was performed using HCS reagent HitKit™ as per manufacturer's instructions (Thermo Fisher Scientific Inc., USA). Briefly, this kit enable to measure cell viability, cell membrane permeability and lysosomal mass/pH, which are toxicity-linked cellular markers. The experimental layout for the automated microscopic analysis, based on the In Cell Analyzer 1000, was composed of untreated cells (negative control or N/T), cells treated with chemically-modified SWCNTs and cells exposed to cisplatin (positive control or P/T), which is a platinum-based cytostatic drug used to treat various types of cancers^40^. Images were acquired in a stereology configuration of five randomly selected fields at 10× magnification using three detection channels with different excitation filters. The rate of cell viability and proliferation were assessed by the automated analysis of the nuclear count and morphology (DAPI filter); in parallel the fluorescent staining intensities reflecting cell permeability (FITC filter) and lysosomal mass/pH changes (TRITC filter) were also quantified for each individual cell present in the examined microscopic fields (IN Cell Investigator, GE Healthcare, UK).

### Cytokines secretion

The expression of pro-inflammatory cytokines (Tumour Necrosis Factor-alpha (TNF-α) and Interleukin-6 (IL-6)) expressed by THP-1 and A549 cells exposed to chemically-treated SWCNTs was quantified by Enzyme-Linked Immunosorbent Assays (ELISAs) (Human TNF-α/TNFSF1A and Human IL-6 DuoSet ELISA kit, R&D Systems, Minneapolis, USA), according to the manufacturer's manual. The assays were carried out in duplicates. The optical density of each well at 450 nm was determined by means of an Epoch microplate reader (Biotek, USA), calibrated against standards and corrected by subtracting the optical aberration of the 96-well plastic plate at 540 nm. Cells were counted using HCSA and Trypan Blue exclusion assay in order to quantify the cytokine production as picograms per cell (pg/cell) at the different exposure concentrations and time points. Cell exposed to LPS were used as positive control (P/T).

### Statistical analysis

A two-way analysis of variance (ANOVA) followed by a Bonferroni post-test analysis was carried out for all HCSA and ELISA assays (Prism; Graph-Pad Software Inc., USA). *p* < 0.05 was considered statistically significant. The entire list of *p* values can be found as Supplementary Tables S1–S22 online. HCSA and ELISA data, as well as the protein citrullination results, are presented as mean values (n_test_ = 2) ± standard error of the mean and normalized to the negative control. Due to the large amount of information acquired by HCSA, a data mining and exploration platform was used (KNIME (http://KNIME.org, 2.0.3) in combination with a HiTS screening module (http://code.google.com/p/hits 0.3.0) in order to screen and normalize all parameters under investigation as previously reported^27^,^36^,^37^,^38^. All measured parameters were normalized using the percentages of the positive controls. Z-score was used for scoring the normalized values. These scores were summarized using the mean function as follows: Z-score = (x-mean)/SD. Heatmaps (i.e., graphical illustration in a colorimetric gradient table format) were adopted as the most suitable schematic representation to report on any statistical significance and variation from normalized controls based on their Z-score value. Heatmap tables illustrate the range of variation of each quantified parameter from the minimum (green), through the mean (yellow), to the maximum (red) value.

## Author Contributions

B.M.M. and A.P.-M. conceptualized and planned the study; B.M.M., D.M. and A.P.-M. carried out HCSA and ELISA experiments, analysed data and wrote the paper; A.K. and D.L. synthesized the chemically-treated SWCNTs; A.M.D. contributed to the optimization of the HCSA protocols; A.P.-M. and Y.V. coordinated the study. All authors discussed the results and commented on the paper.

## Supplementary Material

Supplementary Information Supplementary Information
