# Supplementary material for: Citrullination as early-stage indicator of cell response to Single-Walled Carbon Nanotubes
Source: Sci Rep. 2013 Jan 24;3:1124. doi: 10.1038/srep01124 (PMC3554256; doi:10.1038/srep01124)
Supplement: Supplementary Information [file srep01124-s1.pdf]

Citrullination as early-stage indicator of cell response to Single-Walled Carbon Nanotubes

Bashir Mustafa Mohamed[1, †], Dania Movia[1, 2, †], Anton Knyazev[3], Dominique Langevin[3], Anthony Mitchell Davies[1], Adriele Prina-Mello[1, 2, †, *] and Yuri Volkov[1, 2]

[1]Department of Clinical Medicine, Trinity College Dublin, Ireland.

[2]Centre for Research on Adaptive Nanostructures and Nanodevices, Trinity College Dublin, Ireland.

[3]Universite Paris 11, Laboratoire de Physique des Solides, France.

*Corresponding author: prinamea@tcd.ie

[†]These authors equally contributed to this work.

Supplementary Information

CONTENT

1. ADDITIONAL TABLES page S2
2. ADDITIONAL CHARACTERISATION DATA page S13
3. REFERENCES page S14

Table S1: Statistical significance of the citrullination levels in THP-1 cell cultures exposed to p-SWCNTs, p-SWCNTs/BSA, f-SWCNTs, f-SWCNTs/BSA and Mal-SWCNTs/BSA for 6 h (ANOVA test; *ns*: $p > 0.05$). p value for positive control < 0.001.

Sample	Dose (µg/ml)	p value
p-SWCNTs	1	$p < 0.01$
	5	$p < 0.01$
	10	$p < 0.01$
p-SWCNTs/BSA	1	$p < 0.01$
	5	$p < 0.01$
	10	$p < 0.01$
f-SWCNTs	1	*ns*
	5	*ns*
	10	*ns*
f-SWCNTs/BSA	1	*ns*
	5	*ns*
	10	$p < 0.05$
Mal-SWCNTs/BSA	1	*ns*
	5	$p < 0.01$
	10	$p < 0.001$

Table S2: Statistical significance of the citrullination levels in THP-1 cell cultures exposed to p-SWCNTs, p-SWCNTs/BSA, f-SWCNTs, f-SWCNTs/BSA and Mal-SWCNTs/BSA for 24 h (ANOVA test; *ns*: $p > 0.05$). p value for positive control < 0.001.

Sample	Dose (µg/ml)	p value
p-SWCNTs	1	$p < 0.001$
	5	$p < 0.001$
	10	$p < 0.001$
p-SWCNTs/BSA	1	*ns*
	5	$p < 0.01$
	10	$p < 0.01$
f-SWCNTs	1	*ns*
	5	$p < 0.01$
	10	$p < 0.01$
f-SWCNTs/BSA	1	$p < 0.01$
	5	$p < 0.001$
	10	$p < 0.001$
Mal-SWCNTs/BSA	1	*ns*
	5	$p < 0.01$
	10	$p < 0.001$

Table S3: Statistical significance of the citrullination levels in A549 cell cultures exposed to p-SWCNTs, p-SWCNTs/BSA, f-SWCNTs, f-SWCNTs/BSA and Mal-SWCNTs/BSA for 6 h (ANOVA test; *ns*: $p > 0.05$). p value for positive control < 0.001.

Sample	Dose (μg/ml)	p value
p-SWCNTs	1	*ns*
	5	*ns*
	10	*ns*
p-SWCNTs/BSA	1	$p < 0.05$
	5	$p < 0.01$
	10	$p < 0.01$
f-SWCNTs	1	*ns*
	5	$p < 0.05$
	10	$p < 0.001$
f-SWCNTs/BSA	1	*ns*
	5	*ns*
	10	*ns*
Mal-SWCNTs/BSA	1	*ns*
	5	$p < 0.05$
	10	$p < 0.001$

Table S4: Statistical significance of the citrullination levels in A549 cell cultures exposed to p-SWCNTs, p-SWCNTs/BSA, f-SWCNTs, f-SWCNTs/BSA and Mal-SWCNTs/BSA for 24 h (ANOVA test; *ns*: $p > 0.05$). p value for positive control < 0.001.

Sample	Dose (μg/ml)	p value
p-SWCNTs	1	*ns*
	5	*ns*
	10	$p < 0.01$
p-SWCNTs/BSA	1	$p < 0.01$
	5	$p < 0.001$
	10	$p < 0.001$
f-SWCNTs	1	$p < 0.01$
	5	$p < 0.01$
	10	$p < 0.001$
f-SWCNTs/BSA	1	*ns*
	5	*ns*
	10	$p < 0.01$
Mal-SWCNTs/BSA	1	*ns*
	5	$p < 0.01$
	10	$p < 0.001$

Table S5: Statistical significance of the cell count changes in THP-1 cell cultures exposed to p-SWCNTs, p-SWCNTs/BSA, f-SWCNTs, f-SWCNTs/BSA and Mal-SWCNTs/BSA for 24 h (ANOVA test; *ns*: $p > 0.05$). *p* value for positive control < 0.001.

Sample	Dose (μg/ml)	p value
p-SWCNTs	1	*ns*
	5	$p < 0.01$
	10	$p < 0.01$
p-SWCNTs/BSA	1	*ns*
	5	$p < 0.05$
	10	$p < 0.01$
f-SWCNTs	1	*ns*
	5	$p < 0.01$
	10	$p < 0.01$
f-SWCNTs/BSA	1	$p < 0.01$
	5	$p < 0.01$
	10	$p < 0.01$
Mal-SWCNTs/BSA	1	$p < 0.01$
	5	$p < 0.01$
	10	$P < 0.01$

Table S6: Statistical significance of the cell count changes in A549 cell cultures exposed to p-SWCNTs, p-SWCNTs/BSA, f-SWCNTs, f-SWCNTs/BSA and Mal-SWCNTs/BSA for 24 h (ANOVA test; *ns*: $p > 0.05$). *p* value for positive control < 0.001.

Sample	Dose (μg/ml)	p value
p-SWCNTs	1	*ns*
	5	*ns*
	10	$p < 0.05$
p-SWCNTs/BSA	1	*ns*
	5	*ns*
	10	$p < 0.05$
f-SWCNTs	1	*ns*
	5	*ns*
	10	$p < 0.01$
f-SWCNTs/BSA	1	*ns*
	5	*ns*
	10	$p < 0.01$
Mal-SWCNTs/BSA	1	*ns*
	5	*ns*
	10	$p < 0.01$

Table S7: Statistical significance of the cell membrane permeability changes in THP-1 cell cultures exposed to p-SWCNTs, p-SWCNTs/BSA, f-SWCNTs, f-SWCNTs/BSA and Mal-SWCNTs/BSA for 24 h (ANOVA test; *ns*: $p > 0.05$). *p* value for positive control < 0.001.

Sample	Dose (μg/ml)	p value
p-SWCNTs	1	*ns*
	5	$p < 0.05$
	10	$p < 0.01$
p-SWCNTs/BSA	1	*ns*
	5	*ns*
	10	$p < 0.01$
f-SWCNTs	1	*ns*
	5	$p < 0.05$
	10	$p < 0.01$
f-SWCNTs/BSA	1	*ns*
	5	$p < 0.05$
	10	$p < 0.01$
Mal-SWCNTs/BSA	1	*ns*
	5	*ns*
	10	$p < 0.01$

Table S8: Statistical significance of the cell membrane permeability changes in A549 cell cultures exposed to p-SWCNTs, p-SWCNTs/BSA, f-SWCNTs, f-SWCNTs/BSA and Mal-SWCNTs/BSA for 24 h (ANOVA test; *ns*: $p > 0.05$). *p* value for positive control < 0.001.

Sample	Dose (μg/ml)	p value
p-SWCNTs	1	*ns*
	5	*ns*
	10	*ns*
p-SWCNTs/BSA	1	*ns*
	5	$p < 0.05$
	10	$p < 0.05$
f-SWCNTs	1	*ns*
	5	*ns*
	10	*ns*
f-SWCNTs/BSA	1	*ns*
	5	*ns*
	10	*ns*
Mal-SWCNTs/BSA	1	*ns*
	5	*ns*
	10	*ns*

Table S9: Statistical significance of the lysosomal mass/pH changes in THP-1 cell cultures exposed to p-SWCNTs, p-SWCNTs/BSA, f-SWCNTs, f-SWCNTs/BSA and Mal-SWCNTs/BSA for 24 h (ANOVA test; *ns*: $p > 0.05$). *p* value for positive control < 0.001.

**Sample**	**Dose (µg/ml)**	**p value**
p-SWCNTs	1	*ns*
	5	$p < 0.05$
	10	$p < 0.01$
p-SWCNTs/BSA	1	*ns*
	5	*ns*
	10	$p < 0.01$
f-SWCNTs	1	*ns*
	5	*ns*
	10	$p < 0.01$
f-SWCNTs/BSA	1	*ns*
	5	$p < 0.05$
	10	$p < 0.01$
Mal-SWCNTs/BSA	1	*ns*
	5	$p < 0.01$
	10	$p < 0.01$

Table S10: Statistical significance of the lysosomal mass/pH changes in A549 cell cultures exposed to p-SWCNTs, p-SWCNTs/BSA, f-SWCNTs, f-SWCNTs/BSA and Mal-SWCNTs/BSA for 24 h (ANOVA test; *ns*: $p > 0.05$). *p* value for positive control < 0.001.

**Sample**	**Dose (µg/ml)**	**p value**
p-SWCNTs	1	*ns*
	5	*ns*
	10	$p < 0.05$
p-SWCNTs/BSA	1	*ns*
	5	$p < 0.05$
	10	$p < 0.05$
f-SWCNTs	1	*ns*
	5	*ns*
	10	*ns*
f-SWCNTs/BSA	1	*ns*
	5	*ns*
	10	$p < 0.05$
Mal-SWCNTs/BSA	1	*ns*
	5	$p < 0.01$
	10	$p < 0.01$

Table S11: Statistical significance of the nuclear size changes in THP-1 cell cultures exposed to p-SWCNTs, p-SWCNTs/BSA, f-SWCNTs, f-SWCNTs/BSA and Mal-SWCNTs/BSA for 24 h (ANOVA test; *ns*: $p > 0.05$). p value for positive control < 0.001.

Sample	Dose (µg/ml)	p value
p-SWCNTs	1	*ns*
	5	*ns*
	10	$p < 0.05$
p-SWCNTs/BSA	1	*ns*
	5	*ns*
	10	$p < 0.01$
f-SWCNTs	1	*ns*
	5	*ns*
	10	$p < 0.01$
f-SWCNTs/BSA	1	*ns*
	5	*ns*
	10	*ns*
Mal-SWCNTs/BSA	1	*ns*
	5	$p < 0.05$
	10	$p < 0.01$

Table S12: Statistical significance of the nuclear size changes in A549 cell cultures exposed to p-SWCNTs, p-SWCNTs/BSA, f-SWCNTs, f-SWCNTs/BSA and Mal-SWCNTs/BSA for 24 h (ANOVA test; *ns*: $p > 0.05$). p value for positive control < 0.001.

Sample	Dose (µg/ml)	p value
p-SWCNTs	1	*ns*
	5	*ns*
	10	$p < 0.05$
p-SWCNTs/BSA	1	*ns*
	5	*ns*
	10	$p < 0.05$
f-SWCNTs	1	*ns*
	5	*ns*
	10	*ns*
f-SWCNTs/BSA	1	*ns*
	5	*ns*
	10	*ns*
Mal-SWCNTs/BSA	1	*ns*
	5	$p < 0.05$
	10	$p < 0.01$

Table S13: Statistical significance of the nuclear intensity changes in THP-1 cell cultures exposed to p-SWCNTs, p-SWCNTs/BSA, f-SWCNTs, f-SWCNTs/BSA and Mal-SWCNTs/BSA for 24 h (ANOVA test; *ns*: $p > 0.05$). p value for positive control < 0.001.

**Sample**	**Dose (µg/ml)**	**p value**
p-SWCNTs	1	*ns*
	5	*ns*
	10	$p < 0.01$
p-SWCNTs/BSA	1	*ns*
	5	*ns*
	10	$p < 0.01$
f-SWCNTs	1	*ns*
	5	*ns*
	10	$p < 0.01$
f-SWCNTs/BSA	1	*ns*
	5	$p < 0.05$
	10	$p < 0.01$
Mal-SWCNTs/BSA	1	*ns*
	5	$p < 0.01$
	10	$p < 0.01$

Table S14: Statistical significance of the nuclear intensity changes in A549 cell cultures exposed to p-SWCNTs, p-SWCNTs/BSA, f-SWCNTs, f-SWCNTs/BSA and Mal-SWCNTs/BSA for 24 h (ANOVA test; *ns*: $p > 0.05$). p value for positive control < 0.001.

**Sample**	**Dose (µg/ml)**	**p value**
p-SWCNTs	1	*ns*
	5	*ns*
	10	$p < 0.05$
p-SWCNTs/BSA	1	*ns*
	5	*ns*
	10	$p < 0.05$
f-SWCNTs	1	*ns*
	5	*ns*
	10	$p < 0.05$
f-SWCNTs/BSA	1	*ns*
	5	*ns*
	10	*ns*
Mal-SWCNTs/BSA	1	*ns*
	5	$p < 0.05$
	10	$p < 0.01$

Table S15: Statistical significance of the IL-6 secretion in THP-1 cell cultures exposed to p-SWCNTs, p-SWCNTs/BSA, f-SWCNTs, f-SWCNTs/BSA and Mal-SWCNTs/BSA for 6 h (ANOVA test; *ns*: $p > 0.05$). *p* value for positive control < 0.001.

Sample	Dose (µg/ml)	p value
p-SWCNTs	1	*ns*
	5	*ns*
	10	*ns*
p-SWCNTs/BSA	1	*ns*
	5	$p < 0.001$
	10	$p < 0.01$
f-SWCNTs	1	$p < 0.01$
	5	$p < 0.001$
	10	$p < 0.001$
f-SWCNTs/BSA	1	$p < 0.05$
	5	$p < 0.001$
	10	$p < 0.001$
Mal-SWCNTs/BSA	1	$p < 0.05$
	5	$p < 0.01$
	10	$p < 0.001$

Table S16: Statistical significance of the IL-6 secretion in THP-1 cell cultures exposed to p-SWCNTs, p-SWCNTs/BSA, f-SWCNTs, f-SWCNTs/BSA and Mal-SWCNTs/BSA for 24 h (ANOVA test; *ns*: $p > 0.05$). *p* value for positive control < 0.001.

Sample	Dose (µg/ml)	p value
p-SWCNTs	1	*ns*
	5	$p < 0.001$
	10	$p < 0.001$
p-SWCNTs/BSA	1	$p < 0.001$
	5	$p < 0.001$
	10	$p < 0.001$
f-SWCNTs	1	$p < 0.01$
	5	$p < 0.05$
	10	$p < 0.001$
f-SWCNTs/BSA	1	$p < 0.001$
	5	$p < 0.001$
	10	$p < 0.001$
Mal-SWCNTs/BSA	1	$p < 0.001$
	5	$p < 0.001$
	10	$p < 0.001$

Table S17: Statistical significance of the TNF-α secretion in THP-1 cell cultures exposed to p-SWCNTs, p-SWCNTs/BSA, f-SWCNTs, f-SWCNTs/BSA and Mal-SWCNTs/BSA for 6 h (ANOVA test; *ns*: $p > 0.05$). *p* value for positive control < 0.001.

Sample	Dose (µg/ml)	p value
p-SWCNTs	1	*ns*
	5	*ns*
	10	*ns*
p-SWCNTs/BSA	1	$p < 0.05$
	5	$p < 0.01$
	10	$p < 0.01$
f-SWCNTs	1	*ns*
	5	$p < 0.05$
	10	$p < 0.001$
f-SWCNTs/BSA	1	$p < 0.01$
	5	$p < 0.001$
	10	$p < 0.001$
Mal-SWCNTs/BSA	1	$p < 0.001$
	5	$p < 0.001$
	10	$p < 0.001$

Table S18: Statistical significance of the TNF-α secretion in THP-1 cell cultures exposed to p-SWCNTs, p-SWCNTs/BSA, f-SWCNTs, f-SWCNTs/BSA and Mal-SWCNTs/BSA for 24 h (ANOVA test; *ns*: $p > 0.05$). *p* value for positive control < 0.001.

Sample	Dose (µg/ml)	p value
p-SWCNTs	1	*ns*
	5	$p < 0.001$
	10	$p < 0.001$
p-SWCNTs/BSA	1	$p < 0.001$
	5	$p < 0.001$
	10	$p < 0.001$
f-SWCNTs	1	$p < 0.01$
	5	$p < 0.001$
	10	$p < 0.001$
f-SWCNTs/BSA	1	$p < 0.001$
	5	$p < 0.001$
	10	$p < 0.001$
Mal-SWCNTs/BSA	1	$p < 0.001$
	5	$p < 0.001$
	10	$p < 0.001$

Table S19: Statistical significance of the IL-6 secretion in A549 cells exposed to p-SWCNTs, p-SWCNTs/BSA, f-SWCNTs, f-SWCNTs/BSA and Mal-SWCNTs/BSA for 6 h (ANOVA test; *ns*: $p > 0.05$). p value for positive control < 0.001.

Sample	Dose (μg/ml)	p value
p-SWCNTs	1	*ns*
	5	*ns*
	10	*ns*
p-SWCNTs/BSA	1	*ns*
	5	*ns*
	10	*ns*
f-SWCNTs	1	*ns*
	5	*ns*
	10	*ns*
f-SWCNTs/BSA	1	*ns*
	5	*ns*
	10	*ns*
Mal-SWCNTs/BSA	1	*ns*
	5	*ns*
	10	*ns*

Table S20: Statistical significance of the IL-6 secretion in A549 cells exposed to p-SWCNTs, p-SWCNTs/BSA, f-SWCNTs, f-SWCNTs/BSA and Mal-SWCNTs/BSA for 24 h (ANOVA test; *ns*: $p > 0.05$). p value for positive control < 0.001.

Sample	Dose (μg/ml)	p value
p-SWCNTs	1	*ns*
	5	*ns*
	10	*ns*
p-SWCNTs/BSA	1	*ns*
	5	*ns*
	10	*ns*
f-SWCNTs	1	*ns*
	5	*ns*
	10	*ns*
f-SWCNTs/BSA	1	*ns*
	5	*ns*
	10	*ns*
Mal-SWCNTs/BSA	1	*ns*
	5	*ns*
	10	*ns*

Table S21: Statistical significance of the TNF-α secretion in A549 cells exposed to p-SWCNTs, p-SWCNTs/BSA, f-SWCNTs, f-SWCNTs/BSA and Mal-SWCNTs/BSA for 6 h (ANOVA test; *ns*: $p > 0.05$). p value for positive control < 0.001.

Sample	Dose (µg/ml)	p value
p-SWCNTs	1	*ns*
	5	*ns*
	10	*ns*
p-SWCNTs/BSA	1	*ns*
	5	*ns*
	10	*ns*
f-SWCNTs	1	*ns*
	5	*ns*
	10	*ns*
f-SWCNTs/BSA	1	*ns*
	5	*ns*
	10	*ns*
Mal-SWCNTs/BSA	1	*ns*
	5	*ns*
	10	*ns*

Table S22: Statistical significance of the TNF-α secretion in A549 cells exposed to p-SWCNTs, p-SWCNTs/BSA, f-SWCNTs, f-SWCNTs/BSA and Mal-SWCNTs/BSA for 24 h (ANOVA test; *ns*: $p > 0.05$). p value for positive control < 0.001.

Sample	Dose (µg/ml)	p value
p-SWCNTs	1	*ns*
	5	*ns*
	10	*ns*
p-SWCNTs/BSA	1	*ns*
	5	*ns*
	10	*ns*
f-SWCNTs	1	*ns*
	5	*ns*
	10	*ns*
f-SWCNTs/BSA	1	*ns*
	5	*ns*
	10	*ns*
Mal-SWCNTs/BSA	1	*ns*
	5	*ns*
	10	*ns*

2. ADDITIONAL CHARACTERIZATION DATA

The average profile height was measured by AFM, while the O_2 atomic content of p-SWCNTs was quantified by XPS.[1] Zeta potential measurements were performed on SWCNTs solutions in DI water at neutral pH (pH = 7) by Zetasizer Nanoseries Nano-ZS (Malvern Instruments, UK). As previously described in the literature, the electrophoretic determination of zeta potential was calculated by assuming spherical geometry[2,3] and then applying the Smoluchowski approximation.[4,5] Measurements were carried out in six replicates for each solution. Data are reported as average ± standard deviation. The zeta potential of SWCNTs was negative at neutral pH (Table S23), similar to previously reported findings,[2,4,6,7] and showed that all SWCNTs dispersions were stable with exemption of f-SWCNTs/BSA. It has been reported that the modification of the nanomaterial surface can significantly change the colloidal stability of the dispersions and thus the interaction of nanomaterials with cells.

Table S23. Physico-chemical characterization of SWCNT samples: average atomic force microscopy height measurements, oxygen content and zeta potential in DI water for p-SWCNTs, p-SWCNTs/BSA, f-SWCNTs, f-SWCNTs/BSA and Mal-SWCNTs/BSA.

Sample	Average AFM profile height (nm)	O_2 content (atomic %)	Zeta potential (mV)
p-SWCNTs	0.9-1.2	8	-41.4 ± 1.6
p-SWCNTs/BSA	5.8	n/a	-33.0 ± 3.3
f-SWCNTs	1.5	n/a	-19.7 ± 6.8
f-SWCNTs/BSA	5	n/a	-2.5 ± 1.0
Mal-SWCNTs/BSA	8	n/a	-25.0 ± 4.0

3. REFERENCES

1 Knyazev, A. *et al.* Selective Adsorption of Proteins on Single-Wall Carbon Nanotubes by Using a Protective Surfactant. *Chem. Eur. J.*, DOI: 10.1002/chem.201101182 (2011).

2 Sano, M., Okamura, J. & Shinkai, S. Colloidal nature of single-walled carbon nanotubes in electrolyte solution: The Schulze-Hardy rule. *Langmuir* **17**, 7172-7173, doi:Doi 10.1021/La010698+ (2001).

3 Smith, B. *et al.* Colloidal Properties of Aqueous Suspensions of Acid-Treated, Multi-Walled Carbon Nanotubes. *Environ Sci Technol* **43**, 819-825, doi:Doi 10.1021/Es802011e (2009).

4 Hu, H. *et al.* Influence of the zeta potential on the dispersability and purification of single-walled carbon nanotubes. *The journal of physical chemistry. B* **109**, 11520-11524, doi:10.1021/jp050781w (2005).

5 Movia, D. *et al.* Screening the Cytotoxicity of Single-Walled Carbon Nanotubes Using Novel 3D Tissue-Mimetic Models. *ACS Nano* **5**, 9278–9290 (2011).

6 Saleh, N. B., Pfefferle, L. D. & Elimelech, M. Influence of biomacromolecules and humic acid on the aggregation kinetics of single-walled carbon nanotubes. *Environ Sci Technol* **44**, 2412-2418, doi:10.1021/es903059t (2010).

7 Mamedov, A. A. *et al.* Molecular design of strong single-wall carbon nanotube/polyelectrolyte multilayer composites. *Nat Mater* **1**, 190-194, doi:10.1038/nmat747 (2002).

8 Rivera-Gil, P. *et al.* The Challenge To Relate the Physicochemical Properties of Colloidal Nanoparticles to Their Cytotoxicity. *Accounts of chemical research*, doi:10.1021/ar300039j (2012).
